# De Winter T-wave Pattern in Proximal Left Anterior Descending Artery Occlusion

**DOI:** 10.5811/cpcem.2020.5.47322

**Published:** 2020-07-15

**Authors:** David Gregory, Bryan Wexler, Brent Becker

**Affiliations:** Wellspan York Hospital, Department of Emergency Medicine, York, Pennsylvania

**Keywords:** ECG, de Winter, T-wave, STEMI, AMI

## Abstract

**Case Presentation:**

We describe a case of an acute myocardial infarction with an atypical electrocardiogram showing a de Winter T-wave pattern suggesting the 100% proximal left anterior descending artery occlusion seen on emergent cardiac catheterization.

**Discussion:**

Timely recognition of acute myocardial ischemia is paramount for emergency providers. As highlighted in this case, It is important to be mindful of atypical electrocardiogram findings, such as de Winter T-waves, which suggest acute myocardial ischemia.

## CASE PRESENTATION

**Image 1:**
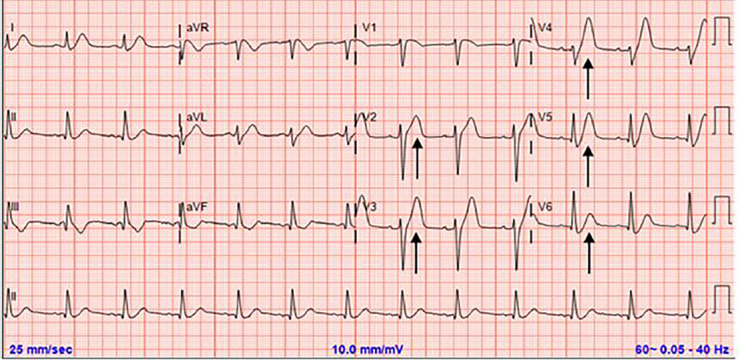
Tall, prominent, symmetric T-waves with associated ST-segment depression in precordial leads (arrows).

A 56-year old male with a history of hypertension presented to the emergency department with one hour of crushing chest pain radiating to the left arm and neck. The symptoms began following exertion, but failed to alleviate with rest. Initial electrocardiogram (ECG) demonstrated hyperacute T-waves with associated ST-segment depression in the precordial leads, consistent with a de Winter T-wave pattern^1^ (Image 1). Due to concern for acute myocardial infarction (AMI), cardiology was consulted for possible percutaneous intervention (PCI).

**Image 2:**
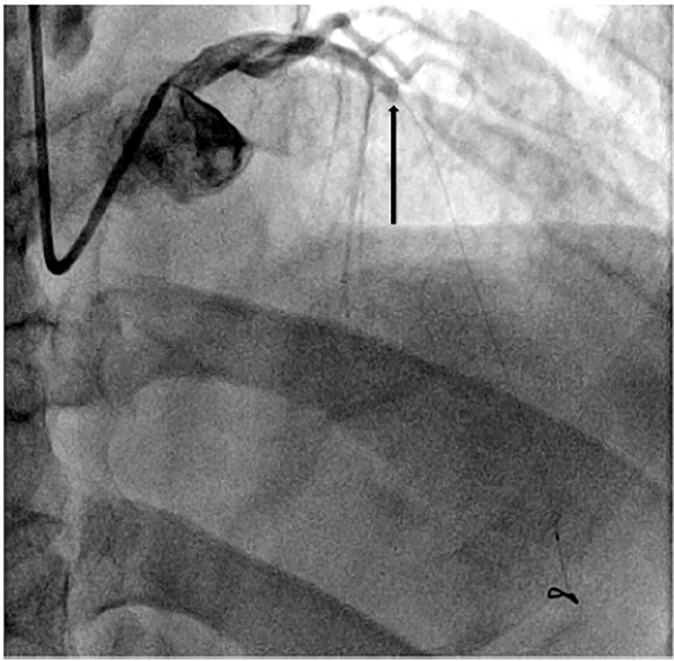
Coronary angiography demonstrating occlusion of the proximal left anterior descending artery (arrow).

Emergent cardiac catheterization was performed and revealed a 100% occlusion of the proximal left anterior descending artery (Image 2). After successful PCI, the patient experienced no further complications and was subsequently discharged on appropriate medical management.

## DISCUSSION

De Winter T-waves are characteristic prominent, symmetric precordial T-waves with associated upsloping ST-segment depression at the J-point.^1^ This ECG pattern lacks traditional ST-segment elevations but is indicative of acute anterior ischemia.^2^ Prior studies suggest approximately 2% of left anterior descending artery occlusions present with de Winter T-waves.^3^

Diagnosis of AMI can be challenging. Emergency physicians are well trained to recognize the pattern of contiguous ST-segment elevations with reciprocal depressions that define traditional criteria for an ST-elevated myocardial infarction (STEMI)^2^; however, clinicians must also be cognizant of atypical ECG findings that suggest acute myocardial ischemia, such as Wellens syndrome, patterns that meet modified Sgarbossa criteria, and as in this case, de Winter T-waves. These less common ECG nuances must be recognized and approached as a STEMI equivalent.

**CPC-EM Capsule**

What do we already know about this clinical entity?

De Winter T-waves are hyperacute precordial T-waves with associated upsloping ST-segment depressions that indicate acute anterior myocardial infarction (MI).

What is the major impact of the image(s)?

The electrocardiogram (ECG) demonstrates the de Winter T-wave pattern that emergency physicians must recognize as a ST-segment elevated MI equivalent.

How might this improve emergency medicine practice?

Recognition of atypical ECG patterns consistent with acute MI can facilitate prompt coronary intervention and salvage at-risk myocardium.
